# John Hopcroft's views on China's education

**DOI:** 10.1093/nsr/nwaa016

**Published:** 2020-02-07

**Authors:** Weijie Zhao, Zhenjiang Hu

**Affiliations:** 1 NSR news editor based, Beijing; 2 Professor and the chair of the Department of Computer Science and Technology at Peking University

## Abstract

Professor John Hopcroft at Cornell University is a Turing Prize winner (1986) and an educator with more than 55 years of teaching experience. For the past 10 years, Hopcroft has been coming to China to give courses to undergraduate students at Shanghai Jiaotong University (SJTU) and has helped SJTU to improve the quality of computer-science education. He also chairs the Center on Frontiers of Computing Studies at Peking University (PKU), the Turing Class at PKU and the Hopcroft Center at Huazhong University of Science and Technology (HUST) in Wuhan, and is engaged in many other projects aiming to upgrade China's computer-science undergraduate education. Recently, *NSR* talked with Professor Hopcroft to learn his views on education in China.

## TRIPS TO CHINA

**NSR:** How long have you been teaching undergraduate courses?

**Hopcroft:** I started teaching in 1964, so I’ve been teaching for over 50 years. I enjoy teaching and research, and that is the reason I haven’t retired yet. Cornell's retirement program is very good. I could have retired on full salary. But I wonder what I would have done if I had retired.

I had very good teachers in the elementary school, high school and college. They really cared about my success. I saw the impact they were having on my life, and I wanted to have that kind of impact on others.

**NSR:** When did you come to China for the first time?

**Hopcroft:** When Americans were first allowed to visit China, I decided to see what China was like. So I went to Macao and crossed the border there, just for curiosity.

For actually being involved in education, it was about 10 years ago when the Chinese Ministry of Education asked me to help them upgrade university education. I worked with 150 faculty members, trying to improve their teaching skills. But I then realized it wasn’t working and suggested that they cancel the project.

Then the president of SJTU approached me. Since then, I have been working with SJTU and have helped them to hire faculty and upgrade the education quality.

**NSR:** How did the Chinese know about you?

**Hopcroft:** I don’t know exactly. But, prior to that, I had worked in about 15 different countries to improve education, including Brazil, Chile, Mexico, Saudi Arabia, Tunisia and India. That may be how China heard about me.

In these countries, I helped a number of students and a few faculty members, but it was difficult to improve the overall quality of education. Now I understand that, unless the top level of government wants to improve education, there's nothing you can do. In China, one of the highest priorities of the Premier is to improve undergraduate education. So there is an opportunity here.

**Figure:**
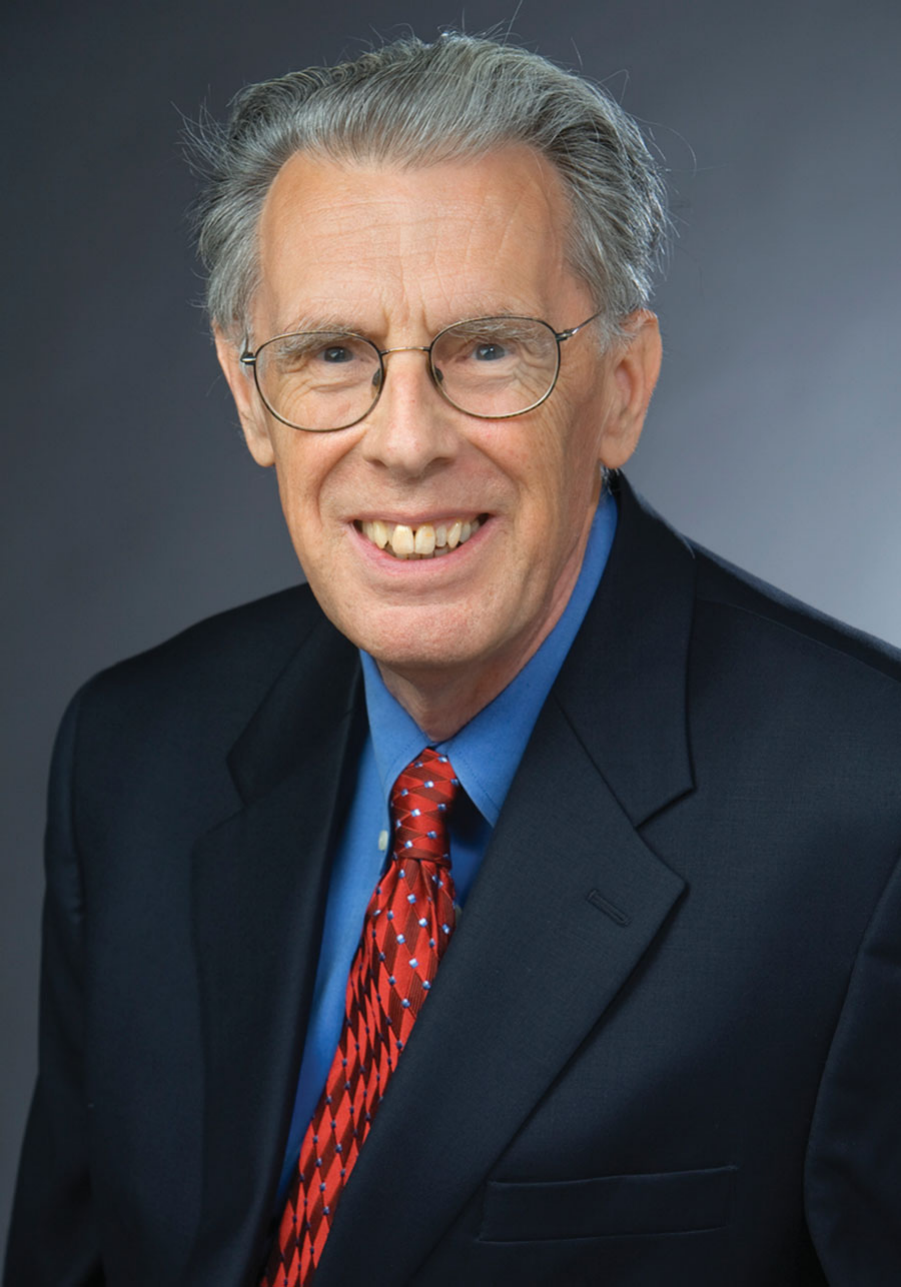
Turing Prize winner, Professor John Hopcroft *(Courtesy of Professor John Hopcroft)*.

## SOME DIFFERENCES BETWEEN THE TWO COUNTRIES

**NSR:** Now you are giving courses to both the American and the Chinese students. What are the differences between these students?

**Hopcroft:** I once brought 30 Chinese students over to the USA for a month. Half of them immediately adopted the US philosophy and curiously started exploring their own research directions, but the other half still would ask me, what do you want me to do today? So one of the major differences is that some of the Chinese students have only been trained to do what they’re told.

I taught the same freshman course at Cornell and at SJTU, and what I realized is that the students at SJTU were better than the students at the top American universities. I think the reason is that talent in the USA is distributed over a fair number of good universities, but in China they all try to get into the top nine universities. So the incoming freshmen at top Chinese universities are better than those at Stanford, Berkeley or Cornell.

But, four years later, when I interviewed these students, the students at Cornell would dominate them. Your university educational system is not working.

Over the past 20 years, Chinese parents realized that their children needed a college degree to get a good job. The number of students going to college increased dramatically and the number of faculty increased from 300 000 to 1 million. You should ask where did they find that many faculty?

**NSR:** We need more high-quality faculty.

**Hopcroft:** That is right. China sends a fair number of students to get PhDs at US universities, but many tend to stay in the USA. I think the reason is that the environment in Chinese universities is not what they want. There is too much pressure to raise research money and publish papers, and many junior faculty have to work for senior faculty. That is not a good environment in which to teach students or to do basic research.

At SJTU, I chaired the committee that was hiring computer-science faculty. However, the new hires were leaving after a couple of years because of the environment. Changing the environment of the entire university was not possible, but the university gave me a piece of the environment for the faculty hired by me. It is working very well now. I have brought back 20 faculty from the USA and we will soon hire 10 more.

**NSR:** What qualities should a good faculty member have?

**Hopcroft:** If our mission is to produce the next generation of talent, then the specific research that the faculty members have done when we hire them is not important. We want that person to stay active during their entire career, so what we look for is somebody who is creative, who is going to keep learning and stay active.

The metrics to evaluate faculty members and university presidents in China should be changed. Increasing the international ranking of the university is the wrong goal since the international rankings are based on research funding and number of papers published. Universities should be evaluated on the quality of the undergraduate program instead of merely on the amount of research funding and the number of publications. There have been changes already and I think the universities in China will improve very rapidly.

When enough talent is trained in China, applied research should be moved out of the university.

—John Hopcroft

There is another difference between universities in the USA and in China. There are enough scientists and engineers in the USA, so if we need to do applied research in order to achieve a civic goal, the companies and institutes can hire the people to do it. Universities are not involved in applied research. They focus on education and basic research. For example, Stanford University set up the Stanford Research Institute in the 1970s and moved applied research off the campus to this independent institution. MIT founded the Lincoln Laboratory for the same purpose.

But, in China, the universities still have to help with applied research. Many senior faculty are actively involved. The problem is that applied research can interfere with education. If the faculty member is teaching the students and also building a company, when the students have a choice of whether they’re going to do some certain research or work for the company, the faculty member is likely to tell them to work for the company. This is a conflict of interest that should not be allowed. China realizes this problem, but it may take 10 to 20 years to make a change. When enough talent is trained in China, applied research should be moved out of the university.

In the USA, the National Science Foundation funds basic research not because they want a specific research project done. It's because they want the faculty member they have funded to produce the next generation of talent. The faculty members are allowed to do research on whatever they are curious about. Actually, it is one of the best investments the USA has ever made.

We do research in all kinds of random directions and occasionally someone does something that creates a whole new industry, providing millions of jobs and billions of dollars to the economy.

**NSR:** Right, and we should combine basic research with education. Both encourage free exploration.

## THE SPECIAL ELITE CLASSES

**NSR:** You have worked for SJTU for about 10 years. What are the outcomes of your efforts?

**Hopcroft:** At SJTU, I chair the John Hopcroft Center for Computer Science, which does not have students but helps to hire faculty for the computer-science department and other programs. We have upped the quality of teaching significantly. A large number of students have gone to US universities and are getting their PhDs. I hope we will eventually upgrade the whole computer-science department.

**NSR:** In recent years, almost all top Chinese universities have set up special elite classes, such as the Zhiyuan College of SJTU, the Turing Class of PKU and the Andrew Yao Class at Tsinghua University. Do you think this is a good movement?

**Hopcroft:** These elite classes are at the equivalent level of top American universities. They are producing world-class students. But there are only 25 or 30 students in each class, so it's not going to solve China's problem. China has to get away from just elite classes to raising the quality of all students. You have so many talented students. If we can expand the elite classes to all of them, Chinese universities will be among the top ones in the world.

**NSR:** Do you think we can extend the elite education mode to graduate education?

**Hopcroft:** Yes. I think once you improve the undergraduate programs and produce better undergraduate students, the quality of the incoming graduate students will improve and the graduate education will improve automatically. But it will take a few years for that to happen.

## BIGGER COMPUTER-SCIENCE DEPARTMENTS ARE NEEDED

**Hopcroft:** I am not sure but I think that, in China, the government determines how many students can go into a given major.

**NSR:** Right.

**Hopcroft:** The difficulty with that is the need for computer-science talent is much bigger than what the country has set. The computer-science departments are not allowed to expand, so the universities are solving this problem by creating other departments such as artificial intelligence (AI), system science and so on.

It is different from the case in the USA, where a department can decide its own size. There are 60 departments at Cornell, and 10% of the total students are in the Department of Computer Science. Students vote with their feet.

The world has gone through an information revolution and we need many students to major in computer science. It is not wise to create multiple new departments, which are actually based on computer science. But maybe we can create a large computer-science college that includes many different departments.

## TO BE MORE FREE AND GLOBALIZED

**NSR:** In the top Chinese universities, many undergraduate students would like to be highly involved in research in the first or second year. Some of them publish in top journals and they are very proud of it. What do you think about that?

**Hopcroft:** Theoretically, I don’t think it's a good experience for an undergraduate to do research. I would encourage them not to, except for one reason: if they want to go on and get a PhD at an American university, they better have a publication. This is unfortunate.

**NSR:** At the University of Tokyo, the students do not belong to any specific department in the first two years. They don’t have much time to publish papers but they try to learn as much as possible and do what they are interested in.

**Hopcroft:** That's very important. I don’t think students going through the university know what they want to do. It would be a major improvement if we admitted students to a college, which could be a college of engineering, or art and sciences, and then let them figure out what they really enjoy one or two years later. Some Chinese universities have begun to do so in certain colleges, but not yet in the whole university.

**NSR:** Some Chinese undergraduate students complain that they have too many courses to learn and little time for a certain one. What about the case in the USA?

**Figure:**
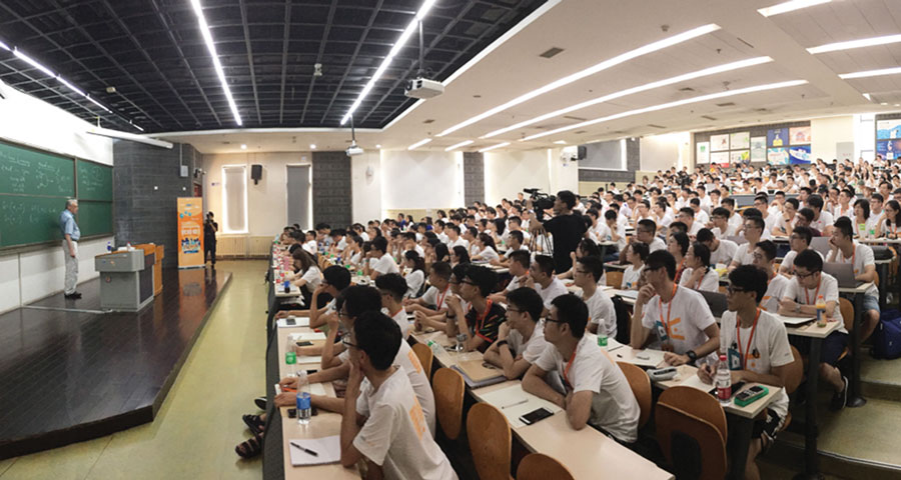
John Hopcroft teaching Chinese students during the 2018 DeeCamp, which is an AI training camp held in Beijing *(Courtesy of Professor John Hopcroft)*.

**Hopcroft:** In the USA, students do not have to take as many courses. They can take a relatively small number and I encourage them to do that. But, for some reason, the students feel that they should take more courses.

Actually, I don’t think taking more courses and staying up late at night mean they are learning more. I think they would learn a lot more if they took fewer courses and could review what was actually taught in those courses. Also they would enjoy their college experience more if they do not have so much work to do.

Both US and Chinese students are taking many courses, actively or passively. But I’m not sure how we’re going to solve that problem.

**NSR:** A top world university must be a globalized university. How can Chinese universities attract more international students?

**Hopcroft:** I think it is very important for a university to be multicultural. There may be several difficulties for Chinese universities to attract foreign students. The language issue is going to be the hardest. Most courses are taught in Mandarin and most students communicate with each other in Mandarin, which makes it difficult for the foreign students. But now translation devices are available to help with the problem. Also, the foreign students may feel isolated. We should set up programs to integrate them with Chinese students.

I think you can approach ministries of education in other countries. Many of them would love to send students to China. In the first several years, it would be hard to attract foreign students. But once you get a few, they will send information back to their home countries through the internet and many more students will come. If you can achieve it, it will definitely enhance the university.

## REWARD THE BEST COMPUTER-SCIENCE TEACHERS

**NSR:** What is the quality of a good teacher?

**Hopcroft:** The most important sign of a good teacher is whether the teacher cares about the success of the students. It is not how much the teacher knows or how good a lecturer a teacher is. I mentioned that I had teachers who really cared about my success. I went to a Catholic elementary school where the teachers had just graduated from high school. They joined the religious order because they wanted to help students and the fact that they didn’t have a college degree was totally unimportant. They wanted to help me learn. They wanted me to have a successful life. That is the most important thing you can evaluate.

**NSR:** The Chinese Ministry of Education proposed a policy that every professor has to give lectures to students. Do you think it's a good action?

**Hopcroft:** To me, the mission of a faculty member is to produce the next generation of talents. So it's crazy to have a faculty member who doesn’t teach. In the USA, we would not allow that. As I mentioned, we should gradually move the faculty members engaged in applied research but do not teach out of the university.

The most important sign of a good teacher is whether the teacher cares about the success of the students.

—John Hopcroft

**NSR:** You proposed a teaching award for the best Chinese undergraduate computer-science teachers in 2018. How did you select the top 26 teachers?

**Hopcroft:** We picked the courses we were going to evaluate, which were the key courses but not the small elite courses. Then we sent two faculty members to sit in two different lectures of a certain course to score it. They evaluated whether the faculty member was knowledgeable about the material, was the material up-to-date, was the faculty member engaging the students, what fraction of the students were listening and what fraction were on their iPhones, and so on. Then we selected and gave awards to the best teachers.

In 2018, we only evaluated courses in the top nine universities. In 2019, we will expand to around 50 universities and give awards to 50 teachers. There are a large number of people involved in this project and we are setting up a nonprofit organization to run it.

Actually, I have permission from the government to evaluate computer-science teaching at the top 50 universities in China. We are sending 45 faculty members around to various universities and will have them actually sit in lectures to score them.

In the first year, 2019, we will keep the ranking result confidential; just tell the university presidents how well they are doing. We want to give them one year's time to improve before we make the 2020 ranking results public. This ranking can help the parents to determine which university to send their children to for the best education.

**NSR:** Who is funding this award?

**Hopcroft:** The top teachers will be awarded around 70 000 RMB. And the project is funded by 10 Chinese companies. I visited these companies and they immediately agreed to fund us because they understood that, if we improve undergraduate education, they’ll be able to hire the talents they need. They view it as a good investment.

## THE SPIRIT OF EDUCATION

**NSR:** In your opinion, what are the missions of education? We should foster which kind of students?

**Hopcroft:** I think what we really want to do is to educate a student to have a good life. Technical education is important for the students to get jobs and for the nation to develop. But we also need to give a broader education including history, sociology and so forth, so that people will be able to make good decisions and deal with all the problems in the ever-changing world.

Also part of education is to help people to enjoy every aspect of life. I don’t believe people can strategically plan their lives. When there's an opportunity to pick one way or another, I think you should pick the one that you’re going to really enjoy. If you have one life to live, you ought to enjoy it.

So I’m opposed to the fact that some of the students are spending too much time studying. When I was in the elementary school, school started at nine o’clock in the morning, and finished at three o’clock in the afternoon with no homework. I enjoyed my elementary school and learned a lot out of the classroom by interacting with other students, playing ball and exploring the city. Elementary-school students in China are very busy. I don’t know whether they enjoy it or not.

**NSR:** Science has developed much in the past half-century. So maybe the students in elementary schools have to learn more things compared with your time.

**Hopcroft:** It is true that there is a tremendous amount of science now, but you are not going to learn it all. If you try to force someone to learn something that they are not capable of learning, it is not going to work. It is more important to learn how to learn and how various disciplines are structured, so that when you need something, you will be able to get access to it.

**NSR:** To foster a good person, which would be the most important educational stage? The elementary school, the high school or the college?

**Hopcroft:** My answer to this question may surprise you. It's none of those. It's the first two years of a child's life.

There have been experiments showing that the first two years are critical for human brain development. Stable environment and good nutrition are needed for the brain to learn how to learn. And, after that, when the children go to elementary school, they will be able to perform well. If you calculate the return on investment, creating a stable environment for every child will be one of the best investments for both the USA and China.

In the USA, we should care about the inner-city areas and try to stabilize the environment for the children there. This would benefit both the children and the nation.

And in China, where both parents are working, many children are cared for by the grandparents. The grandparents may be able to provide enough food, but that is not enough. The children need much more emotional care and early education. When they look up, they want to see someone being around and smile back.

These problems cannot be solved at once. So I will focus on undergraduate education now. After fixing it, I would move to early childhood. It would be an expensive and complex project, but I would like to do something useful.

## FUTURE OF COMPUTER SCIENCE

**NSR:** Are you still doing research work?

**Hopcroft:** Yes. Currently I’m interested in deep learning. People are applying it to many areas, but nobody understands why it works. I want to focus on this question.

Also, it is hard to teach deep learning if it is purely experimental. So I would like to know its theoretical foundations in order to better teach it to my students. That is a special motivation for me.

**NSR:** Many Chinese researchers are applying deep learning in multiple fields, but few of them are studying its theoretical foundations like you. How can this situation be changed?

**Hopcroft:** I think the main problem is that Chinese researchers are evaluated by publications. But if you are going to do basic research, it would be hard to publish.

Actually, I got a tenured position at Princeton University with zero publications in the 1960s. Chinese culture seems to like objective measures such as publication and funding. But if you can build a trustworthy committee that can get rid of these two metrics and evaluate researchers on their creativity and intellectual curiosity, it would greatly benefit basic research in the long run.

**NSR:** Some people say that another AI winter is coming. Do you agree?

**Hopcroft:** I don’t think so because there are so many applications using AI, extending from computer science into many other departments such as medicine, finance and manufacturing. I think, in the future, AI will be a basic technique for everybody, just like mathematics.

But there is one thing I should point out. AI, or deep learning, is not real intelligence; it is pattern recognition at this stage. It is able to do image recognition or translation but it cannot understand the functions and contents of the objects. When we are able to understand function and content, it would be another great revolution that will make AI much more intellectual.

Basic research and free exploration in some crazy directions will bring surprises.

—John Hopcroft

We don’t know how to achieve that goal at this moment, but I believe we can fix it within 40 years. Basic research and free exploration in some crazy directions will bring surprises.

**NSR:** For computer science, what are the other important directions besides AI?

**Hopcroft:** There are a lot of important directions. Security and privacy are going to be major issues. When we use a search engine, it knows a lot about us, from what we like to where we are going. The search engines are free because they are selling our privacy to companies.

Another issue is distributed networks. We used to think of a single computer owned and controlled by a single person. But now there are networks of computers over the entire world and some of the computers are owned by adversaries. These networks are controlling our industries and the question is how to keep them secure. In this area, theories have been developed with the concept of blockchain on top.

Information storage is also important. How can we keep the information in the computers or on the cloud secure? When Microsoft discards its computers with important information, they wipe the memories clean, but also actually shred the computers.

Huge amounts of data on the social networks are also impacting our society. They can affect many issues such as the US election and disease transmission. So the impact of the social networks also needs to be studied.

We are living in the information age, which is totally different from the age of our parents. So there are enormous areas for computer science to explore. AI is an interesting example, but not the whole area.

